# Driving Errors Predicting Pass/Fail On-Road Assessment Outcomes Among Cognitively Impaired Older Drivers

**DOI:** 10.1177/15394492221076494

**Published:** 2022-03-25

**Authors:** Sarah Krasniuk, Diane Mychael, Alexander M. Crizzle

**Affiliations:** 1 University of Saskatchewan, Saskatoon, Canada; 2 St. Joseph’s Health Centre Guelph, Ontario, Canada

**Keywords:** assessment, cognitive impairment, driving, older adults

## Abstract

Older drivers with cognitive impairment (CI)/dementia make significantly more driving errors than healthy controls; however, whether driving errors are predictive of pass/fail outcomes in older drivers with CI/dementia are unclear. This study determined the driving errors that predicted failing an on-road assessment in drivers with CI. We retrospectively collected comprehensive driving evaluation data of 80 participants (76.1 ± 9.3 years) from an Ontario driving assessment center. Adjustment to stimuli (area under the curve [AUC] = 0.88), lane maintenance (AUC = 0.84), and speed regulation errors (AUC = 0.85) strongly predicted pass/fail outcomes. Worse performance on the Trails B (time) and Useful Field of View® (Subtest 2, Subtest 3, and risk index) were significantly correlated with adjustment to stimuli (*p* < .05), lane maintenance (*p* < .05), and speed regulation errors (*p* < .05). Adjustment to stimuli, lane maintenance, and speed regulation errors may be critical indicators of failing an on-road assessment in older drivers with CI. Prioritizing these errors may help identify at-risk drivers.

## Introduction

Older drivers with cognitive impairment (CI)/dementia are at an increased risk of driving impairment (Chee et al., 2017). Compared with healthy older drivers, older drivers with dementia are 2 to 5 times more likely to be involved in a collision (Davis et al., 2018) and 10 times more likely to fail an on-road driving assessment (Chee et al., 2017). Although clinical tests such as the Mini-Mental State Examination (MMSE), Montreal Cognitive Assessment (MoCA), Trail Making Test Part B (Trails B), and Useful Field of View™ (UFOV) are commonly used to screen older drivers with CI (Dickerson et al., 2014; Mathias & Lucas, 2009; Seong-Youl et al., 2014) and in those with and without other medical conditions (Bennett et al., 2016; Hird et al., 2016; Rashid et al., 2020), no clinical test or cut point can solely determine a person’s fitness to drive (Bédard et al., 2008). Due to the ecological validity to real-world driving, the on-road driving assessment may be the most valid measure of a person’s fitness to drive (Bellagamba et al., 2020).

Prior studies show that older adults with CI/dementia make significantly more driving errors than healthy older drivers in adjustment to stimuli, lane maintenance, speed regulation, vehicle positioning, self-directed driving, and their overall on-road driving performance (Barco et al., 2015; Chee et al., 2017; Eramudugolla et al., 2021; Fuermaier et al., 2019; Griffith et al., 2013; Hird et al., 2016; Jacobs et al., 2017; Wadley et al., 2009). Although these studies identify challenging driving situations in older drivers with CI/dementia, they do not specifically examine the types of driving errors that contribute to pass/fail outcomes on the on-road assessment.

Prior studies show that driving errors predict pass/fail outcomes in healthy older drivers (Shechtman et al., 2010) and medical populations, including people diagnosed with Parkinson’s disease (PD; Classen et al., 2014; Devos et al., 2015) and multiple sclerosis (MS; Krasniuk et al., 2017; Krasniuk, Classen, Monahan, et al., 2019; Krasniuk et al., 2020); however, this phenomenon is not well studied in older drivers with CI/dementia. One study found that older drivers with dementia who failed (*n* = 30) an on-road assessment made significantly more dangerous errors (e.g., failure to yield) than those who passed (*n* = 23) when driving straight and making left turns across traffic (i.e., alternatively, when making a right turn if driving on the left side of the road) in moderate to high traffic conditions (e.g., complex intersections, traffic lights, and heavier flow of traffic; Barco et al., 2015).

Understanding the driving errors predictive of failing an on-road assessment may increase the certainty of pass/fail determinations. Knowing the driving errors that predict failing outcomes can help identify deficits associated with CI/dementia and its impact on driving performance and inform potential driving interventions. The purpose of this study was to determine the driving errors critical for failing an on-road assessment in older drivers with CI who were referred by physicians to undergo a comprehensive driving evaluation (CDE). Physicians referred drivers for a CDE if there were concerns regarding cognitive capacity for driving safely. The study objectives were threefold: (a) to determine which driving error(s) differentiated between those who passed and failed the on-road assessment, (b) to examine the associations between clinical test scores and driving errors, and (c) to determine whether driving errors can predict pass/fail on-road outcomes.

## Method

### Study Design

The University’s Research Ethics Board approved this study. The research team retrospectively collected data from CDEs that were performed by an occupational therapist at a driving assessment center in southwestern Ontario. In Ontario, the CDE is administered by an occupational therapist who assesses a person’s fitness to drive through a clinical assessment and an on-road driving assessment that tests the driver’s ability to maneuver a vehicle while detecting, judging, and responding to roadway information in residential, suburban, urban, and highway environments (Canadian Council of Motor Transport Administrators, 2021). The CDE determines whether the driver experiences visual, cognitive, sensory, and/or motor impairments that affect fitness to drive; whether the driver has insight and the ability to compensate or accommodate for such impairments; and whether the driver is compliant with prescribed treatment or existing conditions that may affect fitness to drive (Canadian Council of Motor Transport Administrators, 2021). Determinations include whether the driver is fit to drive and can continue to drive; requires accommodations (e.g., only drive in daylight hours), compensatory strategies (e.g., hand controls to compensate for lower limb impairment), or remedial strategies (e.g., turn head left and right to remediate peripheral field impairment); or is unfit to drive and should cease driving (Canadian Council of Motor Transport Administrators, 2021).

### Participants

Participants were required to undergo a CDE if they were involved in an accident or if a physician was concerned that a medical condition might impact the participants’ driving ability. For this study, the inclusion criteria required drivers to meet the Ministry of Transportation of Ontario’s (2021) vision standards for visual acuity (i.e., binocular score ≤ 20/50 corrected/uncorrected), be referred for a CDE due to cognitive concerns, and that drivers had CI scoring below 26 on the MoCA. Of the 201 drivers, 88 were referred due to cognitive concerns, however, eight were excluded for having MoCA scores of 26 or greater. The sample for this study included 80 drivers. All 80 drivers had complete data for the MoCA and the driving errors in the on-road assessment, except for one participant who was missing data for the number of hazardous errors. Moreover, 79 had complete data for the Trails A, 78 for the Trails B, 72 for the UFOV Subtest 1, 67 for the UFOV Subtest 2, 63 for the UFOV Subtest 3, and 69 for the UFOV risk index (RI).

### Data Collection and Procedures

#### Clinical assessment

The research team collected participants’ demographic information (e.g., age, gender); clinical test scores on the MoCA, Trails A, Trails B, and the UFOV; and the driving outcomes of the on-road assessment (e.g., number of driving errors, pass/fail outcomes).

The MoCA is a measure of general cognitive function with scores from 0 to 30; lower scores indicating worse cognitive function (Nasreddine et al., 2005). A cut point of below 26 on the MoCA has a sensitivity of 89% and a specificity of 75% to detect mild cognitive impairment (MCI; Canadian Task Force on Preventive Health Care, 2016; Nasreddine et al., 2005).

The Trails A is a measure of processing speed and scanning through connecting 25 numbers in ascending order, with lower scores (error and time) indicating better performance (Lezak, 1995). A cut point of 2 or more minutes indicates impairment (Byszewski et al., 2012).

The Trails B is a measure of divided attention through connecting numbers and letters sequentially in ascending order, with lower scores (error and time) indicating better performance. A cut point of 3 or more minutes indicates impairment (Lezak, 1995; Roy & Molnar, 2013).

The UFOV is a computer-based assessment (PC version with touch screen) that measures processing speed (Subtest 1), divided attention (Subtest 2), and selective attention (Subtest 3) in milliseconds; lower scores indicate better performance. Based on performance on each Subtest, the UFOV also computes an overall RI rating from 1 indicating *very low risk* to 5 indicating *high to very high risk* for driving impairment (Ball & Owsley, 1993; Visual Awareness Research Group, 2009).

#### On-road assessment

The on-road driving assessment occurred in a dual-pedal-equipped vehicle with an automatic transmission and was administered by one driving assessor who was an occupational therapist generalist with education in driver rehabilitation. In non-inclement weather conditions, participants took 45 to 60 min to complete the on-road assessment, which consisted of a predetermined route with residential, suburban, urban, and expressway environments. While sitting in the passenger seat, the occupational therapist assessed participants on eight types of maneuvers (i.e., adjustment to stimuli, gap acceptance, lane maintenance, signaling, speed regulation, vehicle positioning, visual scanning, and yielding) during 95 observations that occurred when driving straight (*n* = 47), turning left (*n* = 9), turning right (*n* = 15), and/or changing lanes (*n* = 24). Table 1 provides definitions and examples of errors for each type of driving maneuver. Driving outcomes included the number of errors for each type of driving maneuver and a global rating score with pass, fail, or fail with lessons and retest outcomes. The global rating score was based on participants’ overall performance on the CDE as determined by the occupational therapist. In addition, hazardous errors (e.g., running through stop signs, failing to yield, and crashes) resulted in an immediate failure outcome. For this study, we dichotomized the global rating score into pass/fail outcomes, as done in prior studies (e.g., Classen et al., 2017; Justiss et al., 2006; Shechtman et al., 2010).

**Table 1.** Definition of Driving Maneuvers.

Driving maneuver	Definition	Example of driving error
Adjustment to stimuli	The driver’s response or ability to adjust to critical roadway information (e.g., road sign, traffic light, road user movement, or potential hazards such as potholes), while disregarding redundant information.	Incorrect, impulsive, delayed, or absent responses to roadway information, such as ignoring the posted speed limit.
Gap acceptance	Judging an appropriate safe time or distance to cross in front of or when approaching traffic, such as during unprotected turns.	Over- or underestimating the traveling time or distance to safely cross in front of traffic, such as cutting off opposing traffic or waiting too long to cross traffic.
Lane maintenance	Steering the vehicle to control its lateral or side to side positioning within the lane marking when driving straight, changing lanes, turning, or while stopped.	Parking outside of designated space markings, making wide lane turns by understeering and partially or fully leaving the lane, steering toward the shoulder of the road or other lanes, or encroaching into lanes by oversteering and turning more sharply than intended into oncoming traffic.
Signaling	The proper use and timing of turn signals, including turning them on prior to slowing down for turns, changing lanes, leaving the road, or moving out from a parked position.	Using the wrong signal, turning the signal on too early, turning the signal off too late, or not signaling before or after moving out from a parked position, turning, or changing lanes.
Speed regulation	Controlling or adapting the vehicle’s speed in relation to the posted speed limit or flow of traffic.	Over- or under speeding (e.g., by 10 km or 6 mi), merging too fast or slow based on posted limits, or abruptly or inappropriately braking or accelerating.
Vehicle positioning	Controlling a safe buffer (e.g., 2 s) or distance in front and behind other vehicles when driving straight, during lane changes, and when merging (e.g., following distance).	Traveling too closely behind another vehicle (e.g., tailgating), or providing an inadequate space cushion during lane changes or merges (e.g., other vehicle is in blind spot during a lane change).
Visual scanning	Scanning the environment using head and eye movement to detect or track information (e.g., checking blind spots, observing pedestrians on sidewalks).	Not checking blind spots or side and rearview mirrors during turns, lane changes, merges, or when reversing, or not looking left or right before proceeding through an intersection.
Yielding	Giving the right of way at two- or four-way intersections, when turning right when the traffic light is red (right-hand driving), and when merging into the flow of traffic.	Not giving the right of way or taking a short or long time to turn or merge.

*Note*. The definitions in this table are cited in the literature (e.g., Classen et al., 2017; Justiss et al., 2006; Shechtman et al., 2010).

### Data Analysis

All statistical analyses were performed with Statistical Package for Social Sciences (SPSS; IBM Version 28.0) using a significance level of *p* < .05. Means, standard deviations, and ranges for continuous data, and frequencies and percentages for categorical data summarized participants’ demographic information, clinical test scores, and driving outcomes on the on-road assessment (e.g., driving errors, pass/fail outcomes). Independent sample’s *t* tests examined differences in the number of driving errors made in participants who passed versus failed the on-road assessment. Pearson’s *r* or rank biserial correlations examined the relationships between clinical test scores and driving errors. A logistic regression model with a direct entry and probability of 0.8 determined the predictive relationship between driving errors and pass/fail outcomes. Based on Hosmer et al. (2013) criteria for logistic regression analyses, driving errors with *p* values of ≤.25 from an independent sample’s *t* test were selected as predictor variables. Receiver operating characteristic (ROC) curve with computed area under the curve (AUC) determined the predictive validity of driving errors for predicting pass/fail outcomes (Streiner & Cairney, 2007). A statistically significant AUC of 0.7 or greater indicated that the driving errors provided acceptable discrimination between pass/fail outcomes (Streiner & Cairney, 2007).

## Results

### Sample Characteristics of Older Drivers With CI

The sample included 62 male (78.0%) and 18 female (22.0%) drivers with a mean age of 76.1 years (*SD* = 9.3, range = 45–94 years); 90.0% were 65 years and older. At the time of assessment, 69.0% participants had a valid driver’s license, 12.5% had a 90-day suspension, 17.5% had a temporary license, and 1.0% had a beginner’s license; 19.0% of participants had been advised not to drive prior to their assessment. Participants had been driving for an average of 56.6 years (*SD* = 10.0, range = 29–73 years).

### Clinical Test Scores

The mean MoCA score was 19.6 (*SD* = 4.2, range = 6–25), showing that the sample comprised participants with CI. On average, participants completed the Trails A in 1:10 min (*n* = 79, *SD* = 0:31, range = 0:23–3:21 min) and the Trails B in 5:02 min (*n* = 78, *SD* = 2:44, range = 0:57–13:06 min). On the Trails A, 5.1% of participants took 2 or more minutes to complete the test, indicating deficits in processing speed and scanning. On the Trails B, 71.8% of participants took 3 or more minutes, whereas 42.5% of participants took 5 or more minutes to complete the test, indicating deficits in divided attention. On the UFOV, participants had a mean score of 48.9 ms on Subtest 1 (*n* = 72, *SD* = 66.4, range = 9–310 ms), 278.9 ms on Subtest 2 (*n* = 67, *SD* = 177.1, range = 9–500 ms), and 392.7 ms on Subtest 3 (*n* = 63, *SD* = 136.3, range = 16–500 ms). Participants’ UFOV RI ratings for driving impairment varied: 17.4% with a *very low risk* (RI = 1), 15.9% with a *low risk* (RI = 2), 17.4% with a *low-to-moderate risk* (RI = 3), 23.2% with a *moderate-to-high risk* (RI = 4), and 26.1% with a *high to very high risk* (RI = 5).

### Driving Errors in the On-Road Assessment

On average, participants made 45.2 total driving errors (*SD* = 15.3, range = 7–78) in the on-road assessment. As shown in Table 2, compared with participants who passed the on-road assessment (*n* = 14), participants who failed (*n* = 66) made significantly more adjustment to stimuli, gap acceptance, lane maintenance, speed regulation, visual scanning, and yielding errors. Moreover, 30.0% of those who failed made hazardous errors compared with 0.0% of those who passed. As only 15.0% of participants made gap acceptance errors and 10.0% made yielding errors, we removed these errors from subsequent analyses. Furthermore, as signaling errors had a *p* value of .14 (i.e., Hosmer et al., 2013, criteria *p* ≤.25), we included the error in subsequent analyses.

**Table 2.** The Number of Driving Errors Made by Older Drivers With Cognitive Impairment Who Passed Versus Failed the On-Road Assessment.

Driving errors	Proportion who made errors (%)	Total (*N* = 80)	Fail (*n* = 66)	Pass (*n* = 14)	Statistical significance
Adjustment to stimuli	98.8	4.5 (2.6) 0–11	5.1 (2.5) 1–11	2.0 (1.2) 0–4	*p* **< .0001**
Gap acceptance	15.0	0.2 (0.5) 0–3	0.2 (0.5) 0–3	0.0 (0.0) 0	*p* **= .001**
Lane maintenance	91.3	7.2 (5.4) 0–21	8.2 (5.3) 0–21	2.3 (2.1) 0–7	*p* **< .0001**
Signaling	90.0	3.8 (2.7) 0–11	3.9 (2.5) 0–11	2.8 (3.2) 0–9	*p* = .14
Speed regulation	98.8	9.3 (5.4) 0–22	10.3 (5.2) 3–22	4.3 (2.7) 0–8	*p* **< .0001**
Vehicle positioning	83.8	3.6 (3.0) 0–13	3.7 (3.0) 0–13	2.9 (2.9) 0–10	*p* = .38
Visual scanning	100.0	16.0 (6.1) 3–28	16.8 (5.8) 4–28	12.6 (6.5) 3–27	*p* **= .02**
Yielding	10.0	0.1 (0.5) 0–3	0.2 (0.5) 0–3	0.0 (0.0) 0	*p* **= .01**
Hazardous error	30.0	0.6 (1.5) 0–12	0.7 (1.7) 0–12	0.0 (0.0) 0	*p* **= .002**

*Note*. Driving errors are summarized as mean (standard deviation) and range. The number of participants for hazardous errors is 79.

Statistical significance = *p* < .05, two-tailed. Statistically significant differences between pass/fail groups are highlighted in boldface.

### Correlations Between Clinical Test Scores and the Number of Driving Errors

As presented in Table 3, age and the UFOV Subtest 3 (selective attention) were both significantly and positively correlated with adjustment to stimuli, lane maintenance, speed regulation, and visual scanning errors; the UFOV Subtest 3 also correlated positively with signaling errors. The Trails B, UFOV Subtest 2 (divided attention), and UFOV RI were positively correlated with adjustment to stimuli, lane maintenance, and speed regulation errors; the UFOV Subtest 2 and RI were also correlated positively with hazardous errors. The MoCA was negatively correlated with adjustment to stimuli and lane maintenance errors (e.g., lower [poorer] scores on the MoCA associated with more adjustment and lane errors). The Trails A was correlated positively with lane maintenance errors. The UFOV Subtest 1 (processing speed) was positively correlated with adjustment to stimuli errors.

**Table 3.** Associations Between the Number of Driving Errors and Clinical Assessment Scores of Older Drivers With Cognitive Impairment.

Clinical assessment	Number of driving errors					
	Adjustment to stimuli	Lane maintenance	Signaling	Speed regulation	Visual scanning	Hazardous error
Age (years)	**.4**	**.3**	.2	**.3**	**.4**	.1
MoCA (/30)	**−.4**	**−.2**	**−**.2	**−**.1	**−**.1	**−**.1
Trails A (s)	.1	**.2**	.2	.1	**−**.1	.1
Trails B (s)	**.3**	**.3**	.1	**.2**	.1	.2
UFOV Subtest 1 (ms)	**.2**	.2	.1	.2	**−**.1	.1
UFOV Subtest 2 (ms)	**.4**	**.5**	.2	**.3**	.1	**.3**
UFOV Subtest 3 (ms)	**.4**	**.4**	**.3**	**.4**	**.3**	.2
UFOV RI^a^ (1–5)	**.4**	**.4**	.1	**.3**	.1	**.3**

*Note*. Pearson’s bivariate correlations. Number of participants for correlations with age (*n* = 80); MoCA (*n* = 80); Trails A (*n* = 79); Trails B (*n* = 78); UFOV Subtest 1 (*n* = 72); UFOV Subtest 2 (*n* = 67); UFOV Subtest 3 (*n* = 63); UFOV RI (*n* = 69). MoCA = Montreal Cognitive Assessment; Trails = Trail Making Test; s = second; UFOV = Useful Field of View®; ms = millisecond; RI = risk index.

a Rank biserial correlations.

In boldface: *p* < .05, two-tailed. Statistically significant correlations.

### Driving Errors That Predict Passing Versus Failing an On-Road Assessment

Prior to performing our regression, we assessed for multicollinearity among driving error variables that were significantly different in those who passed and failed the on-road assessment. Adjustment to stimuli was significantly associated with lane maintenance (*r* = 0.3, *p* = .008), signaling (*r* = 0.2, *p* = .04), speed regulation (*r* = 0.4, *p* < .001), and hazardous errors (*r* = 0.3, *p* = .03); lane maintenance was significantly associated with visual scanning (*r* = 0.3, *p* = .01) and hazardous errors (*r* = 0.3, *p* = .009); and speed regulation was significantly associated with signaling (*r* = 0.2, *p* = .04) and visual scanning (*r* = 0.3, *p* = .01). As hazardous errors resulted in immediate failure on the on-road assessment, we removed the variable from further analyses. Furthermore, as the correlational strength was considered low between the driving error variables, all five driving errors (i.e., adjustment to stimuli, lane maintenance, signaling, speed regulation, and visual scanning) were included in the regression model.

The regression model predicting pass/fail outcomes is shown in Table 4. Adjustment to stimuli (odds ratio [OR] = 2.3, *p* = .03), lane maintenance (OR = 1.3, *p* = .05), signaling (OR = 0.9, *p* = .54), speed regulation (OR = 1.4, *p* = .03), and visual scanning errors (OR = 1.0, *p* = .98) collectively predicted pass/fail outcomes in older drivers with CI, with 88.8% accuracy (Nagelkerke *R*^2^ = .7). When removing nonsignificant driving errors from the model (i.e., signaling, visual scanning), adjustment to stimuli (OR = 2.1, *p* = .03, 95% confidence interval [CI] = [1.1, 4.0]), lane maintenance (OR = 1.3, *p* = .04, 95% CI = [1.0, 1.7]), and speed regulation errors (OR = 1.4, *p* = .03, 95% CI = [1.0, 1.9]) collectively predicted pass/fail outcomes with 87.5% accuracy (Nagelkerke *R*^2^ = .7). As adjustment to stimuli, lane maintenance, and speed regulation errors were significant predictors in the model, we compared each of the driving error’s predictive validity with one another.

**Table 4.** Driver Errors as Predictors of Pass/Fail Outcomes on the On-Road Assessment in Older Drivers With Cognitive Impairment.

Number of driving errors	*B*	*SE*	*p* value	OR	95% CI
Adjustment to stimuli	0.8	0.4	**.03**	2.3	[1.1, 4.8]
Lane maintenance	0.3	0.1	**.05**	1.3	[1.0, 1.8]
Signaling	−0.1	0.2	.54	0.9	[0.6, 1.3]
Speed regulation	0.4	0.2	**.03**	1.4	[1.0, 1.9]
Visual scanning	0.0	0.1	.98	1.0	[0.8, 1.2]

*Note. B* = beta or unstandardized regression coefficient, OR = odds ratio, CI = confidence interval for odds ratio.

Statistically significant predictors (*p* < .05, two-tailed) of pass/fail driving outcomes are highlighted in boldface.

**Figure 1.**
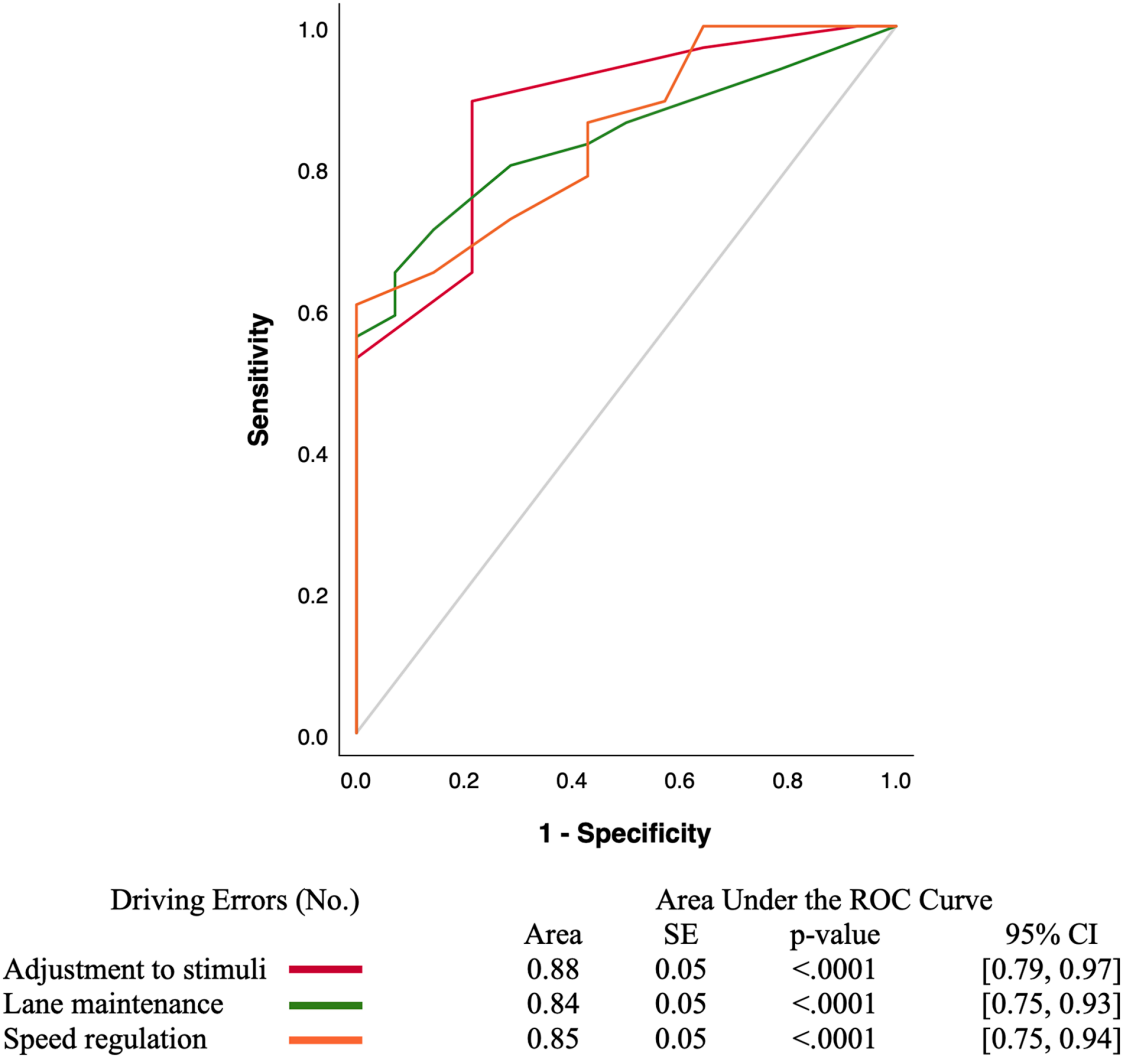
Driving errors predicting pass versus fail outcomes of an on-road assessment in older drivers with CI (*N* = 80, 14 passed, 66 failed). *Note*. CI = confidence interval.

Figure 1 shows the comparisons between adjustment to stimuli, lane maintenance, and speed regulation errors in predicting pass/fail outcomes, which did not significantly differ between one another. As indicated by the AUC, adjustment to stimuli errors predicted 88% (AUC = 0.88, *SE* = 0.05, *p* < .0001, 95% CI = [0.79, 0.97]), lane maintenance errors predicted 84% (AUC = 0.84, *SE* = 0.05, *p* < .0001, 95% CI = [0.75, 0.93]), and speed regulation errors predicted 85% (AUC = 0.85, *SE* = 0.05, *p* < .0001, 95% CI = [0.75, 0.94]) of pass/fail outcomes.

## Discussion

This study determined the driving errors (i.e., adjustment to stimuli, lane maintenance, and speed regulation) critical for failing an on-road assessment in older drivers with CI who, due to cognitive concerns, were referred by physicians to undergo a CDE. Every 1-unit increase in the number of adjustment to stimuli, lane maintenance, or speed regulation errors increased the odds of failing the on-road assessment by 2.1 times, 1.3 times, and 1.4 times, respectively. Furthermore, the strong predictive validity showed that these three driving errors can be used together or individually to predict fail outcomes in older drivers with CI. These findings are consistent with prior studies, which show that older drivers with CI/dementia with losses in memory can become worried, confused, or get lost while driving in more challenging situations than healthy controls, which can contribute to adjustment to stimuli, lane maintenance, and speed regulation errors, as well as more critical events or dangerous situations (Barco et al., 2015; Davis et al., 2018; R. L. Davis & Ohman, 2017; R. L. Davis & Owens, 2021; Feng et al., 2021; Paire-Ficout et al., 2018). Given the strong predictive validity found in our study, driving assessors should prioritize adjustment to stimuli, lane maintenance, and speed regulation errors when assessing fitness to drive in older drivers with CI/dementia.

Our findings are consistent with prior studies, which show that adjustment to stimuli, lane maintenance, and speed regulation errors predict pass/fail on-road outcomes in drivers diagnosed with PD (Classen et al., 2014; Devos et al., 2015), MS (Krasniuk et al., 2017; Krasniuk, Classen, Monahan, et al., 2019; Krasniuk et al., 2020) and dementia (Barco et al., 2015). It is possible that these findings are due to CI being common among these medically at-risk drivers (Fuermaier et al., 2019; Jacobs et al., 2017). However, compared with prior studies, our sample of older drivers with CI had higher failure rates (82.5%) than drivers with PD (e.g., 30%–56%, Classen et al., 2014; Devos et al., 2015) and drivers with MS (e.g., 20%–40%, Krasniuk, Classen, Morrow, et al., 2019). These higher failure rates may be due to our sample having more severe CI as they were referred by physicians for CDEs, whereas most participants in other studies were recruited through tertiary care centers or the community (Classen et al., 2014; Devos et al., 2015; Krasniuk, Classen, Morrow, et al., 2019). Whereas Barco et al. (2015) also recruited older individuals with dementia through physician referral, more participants in our study failed the on-road assessment (82.5% vs. 57%) but showed similar rates of immediate failures due to serious safety concerns or hazardous errors (30% vs. 27%).

Increasing age was more strongly correlated with adjustment to stimuli, lane maintenance, and speed regulation errors compared with the other errors. As all participants had CI, these findings suggest that age, in addition to CI, may contribute to driving performance deficits that may affect a person’s fitness to drive. However, in clinical practice, clients referred to driving assessment centers include individuals who are aging and have CI that affect their driving performance. When assessing these individuals, it would not be possible to isolate the proportion of age-related deficits from CI that contribute to a person’s driving performance. Accordingly, to make our study findings as applicable as possible to clinical practice, we did not control for age in the regression analysis.

The associations between clinical test scores and driving errors show how cognitive deficits are related to driving errors critical for failing the on-road assessment. Worse scores on the MoCA were significantly correlated with adjustment to stimuli and lane maintenance errors, but not with speed regulation, which may be due to all participants having CI and making a high number of speed regulation errors. Although worse scores on the Trails A correlated with lane maintenance errors, and worse scores on the UFOV Subtest 1 correlated with adjustment to stimuli errors, participants’ scores on both tests suggested that Trails A and UFOV Subtest 1 were not challenging to older drivers with CI. Thus, these tests may not identify deficits that may associate with the driving errors critical for failing on-road outcomes. Conversely, worse scores on the Trails B and the UFOV Subtest 2, Subtest 3, and RI were significantly correlated with adjustment to stimuli, lane maintenance, and speed regulation errors. These findings suggest that deficits in processing speed, divided attention, and selective attention may associate with not being able to identify, process, select, or prioritize roadway information that is needed to respond appropriately to the driving environment (i.e., adjustment to stimuli); maintain the side to side positioning of the vehicle when driving straight, changing lanes, or turning (i.e., lane maintenance); or maintain or control speeds, as shown by over- or under speeding in relation to the posted speed limit (i.e., speed regulation). Using the Trails B and UFOV (Subtests 2, 3, and RI) may provide some insight into whether older drivers with CI experience deficits in processing speed and divided/selective attention, which may impact driving performance through making more adjustment to stimuli, lane maintenance, and speed regulation errors. Further research may examine the predictive validity and clinical test cut points of the Trails B and UFOV on predicting adjustment to stimuli, lane maintenance, and speed regulation errors in older drivers with CI.

### Study Limitations

Although participants in this study were referred to undergo a CDE due to cognitive concerns, we could not collect information on whether they had a confirmed diagnosis (e.g., Alzheimer’s disease, other dementias). As this is retrospective data collected from CDEs at a driving assessment center, the missing clinical data may be due to performing poorly on some of the clinical tests and not proceeding onto the more difficult tests (e.g., scoring 500 ms on UFOV Subtest 2 and not proceeding to Subtest 3). During the on-road assessment, we could not determine whether lane maintenance errors involved encroaching or wide lane turns, or whether speed regulation errors involved over- or under speeding. Furthermore, we could not determine the context in which these driving errors occurred (e.g., residential, suburban, urban, or expressway environments). Understanding the complexity (e.g., traffic speed, volume of road users, and flow of traffic) of different driving environments and their influence on driving maneuvers and outcomes may be useful for identifying at-risk drivers or informing/developing driver intervention.

### Implications for Practice

This study shows that adjustment to stimuli, lane maintenance, and speed regulation errors may be critical indicators of failing an on-road assessment in older drivers with CI. Occupational therapists, driving assessors, or other stakeholders who assess driving behaviors may prioritize or weight these errors more heavily to help identify at-risk drivers. The clinical test scores on the Trails B (time for completion) and UFOV (Subtests 2, 3, and RI) correlated significantly with the three driving errors, indicating that poorer performance in divided/selective attention associates with making more adjustment to stimuli, lane maintenance, or speed regulation errors in the on-road assessment. As interventions aimed at promoting the mobility and safety of older drivers need to be designed based on challenging driving situations, occupational therapists or others involved in driver rehabilitation may consider interventions that compensate or remediate for divided/selective attention and other difficulties in adjustment to stimuli, lane maintenance, or speed regulation.

### Future Research

Further research may examine the clinical indicators of poor driving outcomes (i.e., pass/fail, driving errors) through stratifying participants by diagnosis (e.g., MCI, Alzheimer’s disease, and other dementias) and disease severity level (e.g., mild, moderate, and severe). In addition, future research should consider the influence of different driving contexts (e.g., environments, traffic situations) and consider examining the severity or weight as well as the number of driving errors that predict on-road outcomes in older drivers with CI.

## Conclusion

This study found that, individually, adjustment to stimuli, lane maintenance, and speed regulation errors significantly differentiated between passing/failing an on-road assessment in older drivers with CI who, due to cognitive concerns, were referred by physicians to undergo a CDE. When combined, these three driving errors significantly predicted failing outcomes. The significant correlations between clinical tests and these three driving errors suggest that the Trails B and UFOV (Subtests 2, 3, and RI) may be useful in identifying older drivers with CI who may have trouble with adjustment to stimuli, lane maintenance, and/or speed regulation maneuvers. During an on-road assessment, prioritizing or weighting adjustment to stimuli, lane maintenance, and speed regulation errors more heavily may be useful for identifying at-risk older drivers with CI.

## References

[bibr1-15394492221076494] Ball K. Owsley C. (1993). The useful field of view test: A new technique for evaluating age-related declines in visual function. Journal of the American Optometric Association, 64(1), 71–79. 8454831

[bibr2-15394492221076494] Barco P. P. Baum C. M. Ott B. R. Ice S. Johnson A. Wallendorf M. Carr D. B. (2015). Driving errors in persons with dementia. Journal of the American Geriatrics Society, 63(7), 1373–1380. 10.1111/jgs.13508 26140521

[bibr3-15394492221076494] Bédard M. Weaver B. Darzins P. Porter M. M. (2008). Predicting driving performance in older adults: We are not there yet! Traffic Injury Prevention, 9(4), 336–341. 10.1080/15389580802117184 18696390

[bibr4-15394492221076494] Bellagamba D. Vionnet L. Margot-Cattin I. Vaucher P. (2020). Standardized on-road tests assessing fitness-to-drive in people with cognitive impairments: A systematic review. PLOS ONE, 15(5), Article e0233125. 10.1371/journal.pone.0233125 PMC7233547 32421733

[bibr5-15394492221076494] Bennett J. M. Chekaluk E. Batchelor J. (2016). Cognitive tests and determining fitness to drive in dementia: A systematic review. Journal of the American Geriatrics Society, 64(9), 1904–1917. 10.1111/jgs.14180 27253511

[bibr6-15394492221076494] Byszewski A. Molnar F. J. Merkley V. F. Ellen R. L. B. (2012). Driving and dementia toolkits for health professionals and for patients and caregivers. CGS Journal of CME, 2(3), 10–13.

[bibr7-15394492221076494] Canadian Council of Motor Transport Administrators. (2021). National safety code standard 6: Determining driver fitness in Canada Part 1: A model for the administration of driver fitness programs part 2: CCMTA medical standards for drivers. https://www.ccmta.ca

[bibr8-15394492221076494] Canadian Task Force on Preventative Health Care. (2016). Recommendations on screening for cognitive impairment in older adults. Canadian Medical Association Journal, 188(1), 37–46. 10.1503/cmaj.141165 26622001 PMC4695353

[bibr9-15394492221076494] Chee J. N. Rapoport M. J. Molnar F. Herrmann N. O’Neill D. Marottoli R. Mitchell S. Tant M. Dow J. Ayotte D. Lanctot K. L. McFadden R. Taylor J. P. Donaghy P. C. Olsen K. Classen S. Elzohairy Y. Carr D. B. (2017). Update on the risk of motor vehicle collision or driving impairment with dementia: A collaborative international systematic review and meta-analysis. American Journal of Geriatric Psychiatry, 25(12), 1376–1390. 10.1016/j.jagp.2017.05.007 28917504

[bibr10-15394492221076494] Classen S. Brumback B. Monahan M. Malaty I. I. Rodriguez R. L. Okun M. S. McFarland N. R. (2014). Driving errors in Parkinson’s disease: Moving closer to predicting on-road outcomes. American Journal of Occupational Therapy, 68(1), 77–85. 10.5014/ajot.2014.008698 PMC3871971 24367958

[bibr11-15394492221076494] Classen S. Krasniuk S. Alvarez L. Monahan M. Morrow S. A. Danter T. (2017). Development and validity of Western University’s on-road assessment. OTJR: Occupation, Participation and Health, 37(1), 14–29. https://doi.org/10.1177%2F1539449216672859 27744272 10.1177/1539449216672859

[bibr12-15394492221076494] Davis J. D. Wang S. Festa E. K. Luo G. Moharrer M. Bernier J. Ott B. R. (2018). Detection of risky driving behaviors in the naturalistic environment in healthy older adults and mild Alzheimer’s disease. Geriatrics (Basel), 3(2), Article 13. 10.3390/geriatrics3020013 PMC5889300 29632868

[bibr13-15394492221076494] Davis R. L. Ohman J. M. (2017). Driving in early-stage Alzheimer’s disease: An integrative review of the literature. Research in Gerontological Nursing, 10(2), 86–100. 10.3928/19404921-20160920-02 27665752

[bibr14-15394492221076494] Davis R. L. Owens M. (2021). Self-regulation of driving behaviors in persons with early-stage Alzheimer’s disease. Journal of Gerontological Nursing, 47(1), 21–27. 10.3928/00989134-20201209-01 33377981

[bibr15-15394492221076494] Devos H. Ranchet M. Akinwuntan A. E. Uc E. Y. (2015). Establishing an evidence-base framework for driving rehabilitation in Parkinson’s disease: A systematic review of on-road driving studies. NeuroRehabilitation, 37(1), 35–52. 10.3233/NRE-151239 26409692

[bibr16-15394492221076494] Dickerson A. E. Meuel D. B. Ridenour C. D. Cooper K. (2014). Assessment tools predicting fitness to drive in older adults: A systematic review. American Journal of Occupational Therapy, 68(6), 670–680. 10.5014/ajot.2014.011833 25397762

[bibr17-15394492221076494] Eramudugolla R. Huque M. H. Wood J. Anstey K. J. (2021). On-road behavior in older drivers with mild cognitive impairment. Journal of the American Medical Directors Association, 22(2), 399–405.e391. 10.1016/j.jamda.2020.05.046 32698991

[bibr18-15394492221076494] Feng Y. R. Meuleners L. Stevenson M. Heyworth J. Murray K. Fraser M. Maher S. (2021). Driving exposure, patterns and safety critical events for older drivers with and without mild cognitive impairment: Findings from a naturalistic driving study. Accident Analysis & Prevention, 151, Article 105965. 10.1016/j.aap.2020.105965 33429206

[bibr19-15394492221076494] Fuermaier A. B. M. Piersma D. de Waard D. Davidse R. J. de Groot J. Doumen M. J. A. Bredewoud R. A. Claesen R. Lemstra A. W. Scheltens P. Vermeeren A. Ponds R. Verhey F. De Deyn P. P. Brouwer W. H. Tucha O. (2019). Driving difficulties among patients with Alzheimer’s disease and other neurodegenerative disorders. Journal of Alzheimer’s Disease, 69(4), 1019–1030. 10.3233/JAD-181095 31045516

[bibr20-15394492221076494] Griffith H. R. Okonkwo O. C. Stewart C. C. Stoeckel L. E. Hollander J. A. Elgin J. M. Harrell L. E. Brockington J. C. Clark D. G. Ball K. K. Owsley C. Marson D. C. Wadley V. G. (2013). Lower hippocampal volume predicts decrements in lane control among drivers with amnestic mild cognitive impairment. Journal of Geriatric Psychiatry and Neurology, 26(4), 259–266. 10.1177/0891988713509138 24212246 PMC4114386

[bibr21-15394492221076494] Hird M. A. Egeto P. Fischer C. E. Naglie G. Schweizer T. A. (2016). A systematic review and meta-analysis of on-road simulator and cognitive driving assessment in Alzheimer’s disease and mild cognitive impairment. Journal of Alzheimer’s Disease, 53(2), 713–729. 10.3233/jad-160276 27176076

[bibr22-15394492221076494] Hosmer J. D. W. Lemeshow S. Sturdivant R. X. (2013). Applied logistic regression (3rd ed.). Wiley.

[bibr23-15394492221076494] Jacobs M. Hart E. P. Roos R. A. C. (2017). Driving with a neurodegenerative disorder: An overview of the current literature. Journal of Neurology, 264(8), 1678–1696. 10.1007/s00415-017-8489-9 28424901 PMC5533843

[bibr24-15394492221076494] Justiss M. D. Mann W. C. Stav W. Velozo C. (2006). Development of a behind-the-wheel driving performance assessment for older adults. Topics in Geriatric Rehabilitation, 22(2), 121–128. 10.1097/00013614-200604000-00004

[bibr25-15394492221076494] Krasniuk S. Classen S. Monahan M. Danter T. He W. Rosehart H. Morrow S. A. (2019). A strategic driving maneuver that predicts on-road outcomes in adults with multiple sclerosis. Transportation Research Part F, 60, 147–156. 10.1016/j.trf.2018.10.014

[bibr26-15394492221076494] Krasniuk S. Classen S. Morrow S. A. He W. (2020). Driving environments that influence on-road outcomes in persons with Multiple Sclerosis. Transportation Research Part F, 70, 191–198. 10.1016/j.trf.2020.03.003

[bibr27-15394492221076494] Krasniuk S. Classen S. Morrow S. A. Monahan M. Danter T. Rosehart H. He W. (2017). Driving errors that predict on-road outcomes in adults with multiple sclerosis. OTJR: Occupation, Participation and Health, 37(3), 115–124. 10.1177/1539449217708554 28539098

[bibr28-15394492221076494] Krasniuk S. Classen S. Morrow S. A. Tippett M. Knott M. Akinwuntan A. (2019). Clinical determinants of fitness to drive in persons with Multiple Sclerosis: Systematic review. Archives of Physical Medicine and Rehabilitation, 100(8), 1534–1555. 10.1016/j.apmr.2018.12.029 30690007

[bibr29-15394492221076494] Lezak M. D. (1995). Neuropsychological Assessment (3rd ed.). Oxford University Press.

[bibr30-15394492221076494] Mathias J. L. Lucas L. K. (2009). Cognitive predictors of unsafe driving in older drivers: A meta-analysis. International Psychogeriatrics, 21(4), 637–653. 10.1017/S1041610209009119 19470197

[bibr31-15394492221076494] Ministry of Transportation of Ontario. (2021). Medical review for Ontario drivers. https://www.ontario.ca/page/medical-review-ontario-drivers

[bibr32-15394492221076494] Nasreddine Z. S. Phillips N. A. Bedirian V. Charbonneau S. Whitehead V. Collin I. Cummings J. L. Chertkow H. (2005). The Montreal cognitive assessment, MoCA: A brief screening tool for mild cognitive impairment. Journal of the American Geriatrics Society, 53(4), 695–699. 10.1111/j.1532-5415.2005.53221.x 15817019

[bibr33-15394492221076494] Paire-Ficout L. Lafont S. Conte F. Coquillat A. Fabrigoule C. Ankri J. Blanc F. Gabel C. Novella J. L. Morrone I. Mahmoudi R. (2018). Naturalistic driving study investigating self-regulation behavior in early Alzheimer’s disease: A pilot study. Journal of Alzheimer’s Disease, 63(4), 1499–1508. 10.3233/JAD-171031 29782312

[bibr34-15394492221076494] Rashid R. Standen P. Carpenter H. Radford K. (2020). Systematic review and meta-analysis of association between cognitive tests and on-road driving ability in people with dementia. Neuropsychological Rehabilitation, 30(9), 1720–1761. 10.1080/09602011.2019.1603112 31018100

[bibr35-15394492221076494] Roy M. Molnar F. (2013). Systematic review of the evidence for Trails B cut-off scores in assessing fitness-to-drive. Canadian Geriatrics Journal, 16(3), 120–142. 10.5770/cgj.16.76 23983828 PMC3753211

[bibr36-15394492221076494] Seong-Youl C. Jae-Shin L. A-Young S. (2014). Cognitive test to forecast unsafe driving in older drivers: Meta-analysis. NeuroRehabilitation, 35(4), 771–778. 10.3233/NRE-141170 25318777

[bibr37-15394492221076494] Shechtman O. Awadzi K. D. Classen S. Lanford D. N. Joo Y. (2010). Validity and critical driving errors of on-road assessment for older drivers. American Journal of Occupational Therapy, 64(2), 242–251. 10.5014/ajot.64.2.242 20437911

[bibr38-15394492221076494] Streiner D. L. Cairney J. (2007). What’s under the ROC? An introduction to receiver operating characteristics curves. The Canadian Journal of Psychiatry, 52(2), 121–128. 10.1177/070674370705200210 17375868

[bibr39-15394492221076494] Visual Awareness Research Group. (2009). UFOV(TM) User’s guide(R). Visual Awareness Research Group.

[bibr40-15394492221076494] Wadley V. G. Okonkwo O. Crowe M. Vance D. E. Elgin J. Ball K. K. Owsley C. (2009). Mild cognitive impairment and everyday function: An investigation of driving performance. Journal of Geriatric Psychiatry and Neurology, 22(2), 87–94. 10.1177/0891988708328215 19196629 PMC2832580

